# *In vivo* two-photon microscopy of the human eye

**DOI:** 10.1038/s41598-019-46568-z

**Published:** 2019-07-12

**Authors:** Francisco J. Ávila, Adrián Gambín, Pablo Artal, Juan M. Bueno

**Affiliations:** Laboratorio de Óptica, Instituto Universitario de Investigación en Óptica y Nanofísica, Universidad de Murcia, Campus de Espinardo (Ed. 34), 30100 Murcia, Spain

**Keywords:** Medical research, Multiphoton microscopy

## Abstract

Two-photon (2P) microscopy is a powerful tool for imaging and exploring label-free biological tissues at high resolution. Although this type of microscopy has been demonstrated in *ex vivo* ocular tissues of both humans and animal models, imaging the human eye *in vivo* has always been challenging. This work presents a novel compact 2P microscope for non-contact imaging of the anterior part of the living human eye. The performance of the instrument was tested and the maximum permissible exposure to protect ocular tissues established. To the best of our knowledge, 2P images of the *in vivo* human cornea, the sclera and the trabecular meshwork are shown for the very first time. Acquired images are of enough quality to visualize collagen arrangement and morphological features of clinical interest. Future implementations of this technique may constitute a potential tool for early diagnosis of ocular diseases at submicron scale.

## Introduction

The eye is the sense organ responsible for vision. Among its physiological complex elements, two of them are rich in collagen: the cornea and the sclera. The former is transparent and contributes to approximately two-thirds of the eye’s refractive power^1^. Within the cornea, the stroma is mainly composed of stacked collagen lamellae and it makes up ~90% of its thickness. Changes in the corneal shape and/or in its mechanical properties might lead to a loss of transparency and in consequence, to vision loss or sight impaired. Then, understanding the morphology of the collagen structural distribution is of great importance in disease diagnosis. On the other hand, the sclera is an opaque structure (known as the white of the eye) that protects and provides rigidity and structural integrity to the ocular globe.

Different clinical instruments are used to image corneal structures. These include specular microscopy (or reflectance confocal microscopy) and optical coherence tomography (OCT)^2,3^. OCT and confocal microscopy are effective when imaging the microstructures of the different corneal layers (epithelial and endothelial cells, keratocytes,…). However, the visualization of the collagen fibers of the stroma has been challenging. In confocal imaging, the performance is limited by the reduced image contrast mechanism from the transparent stromal fibers. Stromal structure can be imaged with OCT, but micrometric resolution is not achievable.

These constraints have been overpassed by using two-photon (2P) microscopy^4^. This is a well-established imaging technique used to study non-labeled biological tissues. It consists on a quasi-simultaneous absorption of two infrared photons, followed by the emission of a unique photon in the visible range. This nonlinear process only occurs when the photon flux of the excitation light is in the range of 10^20^–10^30^ photons/(cm^2^·s). This energy density can be reached if a pulsed (~80 MHz) ultra-short (∼100 fs) laser system is used^4,5^. Since this excitation occurs just within the focal plane, the technique provides inherent confocality. Apart from this intrinsic sectioning capability, penetration depth, minimized photo-bleaching and photo-toxicity effects are also important advantages of this type of microscopy.

2P microscopy includes two-photon excitation fluorescence (TPEF) and second harmonic generation (SHG), among others. TPEF signal arises from flavins and intrinsic chromophores such as NAD(P)H^6^. On the other hand, SHG allows the visualization of non-centrosymmetric structures such as type-I fibrillar collagen^7^.

Since its invention more than 25 years ago, 2P microscopy has been used in many different fields of life sciences^7–10^. However, its successful application to visualize ocular structures made scientists and ophthalmologists increase their interest in this tool (see ref.^11^ as a general reference). Its capability to image the eye’s structures with sub-micron resolution provide potential advantages compared to other “classical” clinical imaging techniques, such as OCT, fluorescence microscopy or confocal reflectance.

Different *ex vivo* human ocular structures such as the retina^12,13^, the sclera^14^, and the trabecular meshwork^15,16^ have been investigated through 2P microscopy. However, much effort and interest were put on the cornea since its early SHG visualization^17^. Although SHG images of the healthy human corneal stroma have been reported by different authors^18–22^, the analysis of pathological corneas raised special attention. In human donor corneas, these pathologies include keratoconus^23–29^, keratitis^29,30^, bullous keratopathy^31^ and fibrosis^32^. More recently, multiple *ex vivo* 2P experiments included evaluation of the feasibility for transplantation of human corneas^33^, analysis of stromal striae^34^ and scars^29^, control of limbal epithelial stem cells in cultured human donor corneas^35^ and objective analysis of corneal structures using non-reference image quality assessment methods^36^.

Although 2P microscopy is useful to understand the changes suffered by corneal collagen under different circumstances, all those previous experiments used exclusively *ex vivo* samples (from human donors). Some previous experiments involved living animal models^37–40^, however not all them were successful providing SHG images of non-labeled corneas.

To the best of our knowledge, 2P imaging microscopy of the living human eye has never been performed before. In this work we present a new compact clinically-adapted 2P microscope to record images of the *in vivo* human eye in a non-contact manner (i.e. using a non-immersion objective). The performance of this customized instrument was tested. Images from different *in vivo* ocular tissues of the anterior segment of human eye were acquired (sclera, trabecular meshwork and cornea). Moreover, the safety limits and the threshold to avoid ocular tissue damage while 2P imaging were also established.

## Results

### Safety considerations for the cornea and the retina during 2P imaging

The main goal of the instrument here described has been to image *in vivo* human ocular tissues. Then, to protect those biological structures, the corresponding irradiance limits have to be established. A high-repetition (or mode-locked) infrared laser system was used as illumination source (see Methods section). This might cause thermal damage in the tissues when the laser power density is above the maximum permissible exposure (MPE) for a given exposure time. Due to this, safe 2P imaging performance of the living human eye requires a well-defined and accurate calculation of the damage threshold. To establish these safety limits for the ocular structures, both the International Commission on Non-Ionizing Radiation Protection (ICNRP)^41^ and the ANSI Z136.1-2000^42^ standards were considered.

The calculation of these limits was performed considering the worst possible scenario: a static focal point on the sample, that is, a confined light spot illuminating the same spatial location during the exposure time corresponding to the acquisition of one image. This assumption benefits the security levels, as the position of the laser beam during imaging recording is not static, but dynamic (that is, the beam scans the ocular tissue during that time).

**Figure 1:**
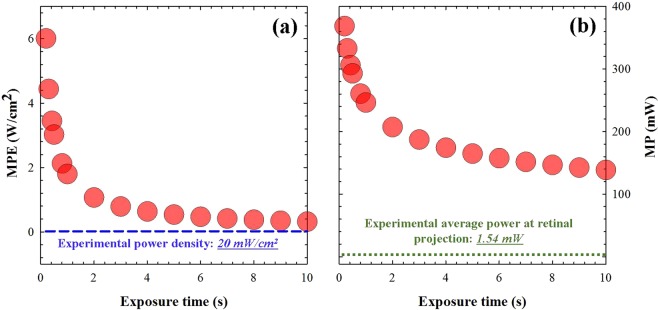
(**a**) MPE as a function of exposure time to protect the cornea during 2P imaging. (**b**) Maximum laser power to protect the human retina as a function of the exposure time (this was computed for a fixed retinal area of 7.6 mm^2^; see details in the text).

Figure 1a depicts the MPE at the corneal plane (i.e. maximum corneal irradiance) as a function of the exposure time, according to the ICNRP. Then, if we consider a static beam focused on the sample during a time equivalent to the image acquisition time (0.42 s), the MPE will be 3.49 W/cm^2^. It must be remembered (see Methods and Suppl. Material) that 0.42 s is the exposure time used to acquire a 2P image of 200 × 200 pixels with a resolution of 1.5 μm/pixel. Since the incident average laser power density at the corneal plane used here was 20 mW/cm^2^ (see Suppl. Material for details on this), the experimental settings lead to conditions well below the MPE. For a better understanding, this power density is represented with a dotted blue line in Fig. 1a.

Although the instrument has been designed to acquire 2P images of the anterior surface of the eye, it is also important to consider that the laser beam, after focusing on the cornea, it propagates across the ocular media, reaching the retina. This light spot on the retina must also be within the safety limits.

To calculate the retinal projection, a simplified model of both the microscope optics and the eye has been simulated using an optical design software (Zemax LCC, Kirkland, WA, USA). The eye model was composed of cornea, aqueous humor, lens, vitreous and retina. Data of dimensions, refractive indices and curvatures were taken from the literature^43^. Ocular absorption was considered to be null.

Considering once again a static laser focused on the cornea, the size of the retinal projection was 7.6 mm^2^. For this retinal area, and according the ANSI Z136.1-2000 standards, the absolute maximum laser power (MP) to avoid retinal damage as a function of the exposure time is shown in Fig. 1b. For an exposure time of 0.42 s, this MP corresponds to 306 mW. This threshold corresponds to a corneal power density of 4 W/cm^2^, a value which is two orders of magnitude higher than the 20 mW/cm^2^ used in this experiment. In addition, for this corneal illumination value, the total laser power delivered to the retinal projection is 1.54 mW (200 times lower than the MP above computed, 306 mW). For the sense of completeness, this value has also been plotted in Fig. 1b. All these calculations corroborate that the experimental conditions here used are below the damage threshold, and both the cornea and the retina are protected during 2P imaging operation.

### SHG images of living human ocular structures

**Figure 2:**
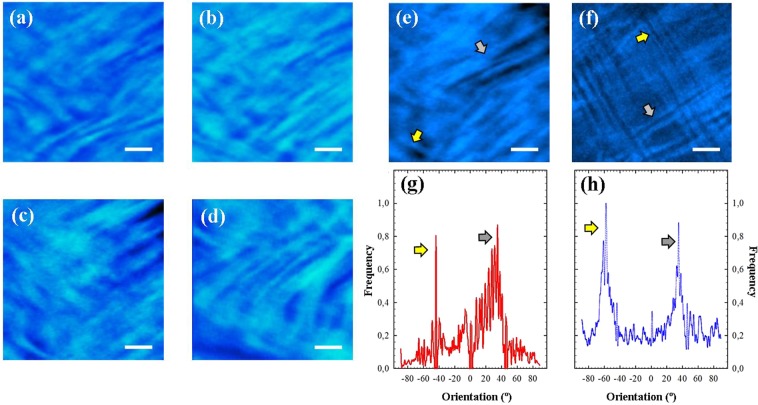
(**a**–**d**) SHG images of the *in vivo* corneal stroma (apex region) of two different subjects (#1 (a, c); #2 (**b**,**d**) and two different depth locations: (**a**,**b**) anterior stroma ~50-µm depth; (**c**,**d**) posterior stroma ~350-µm depth. (**e**,**f**) Comparison of SHG images of the corneal stroma recorded in *in vivo* and *ex vivo* conditions, where the spatial pattern could be clearly visualized. The arrows indicate the two POs. Scale bar: 50 µm. The image corresponding to the *ex vivo* sample was recorded as explained in ref.^21^. (**g**,**h**) PO histograms showing the two orientations of the fibers (peaks). Each *in vivo* SHG image corresponds to an individual frame (image averaging was not used). Scale bar: 50 µm.

Figure 2a-2d show SHG images of the living human cornea from two healthy subjects (subject #1, 2a and 2c; subject #2, 2b and 2d). Images were acquired at the corneal apex and correspond to two different stroma locations: anterior (Fig. 2a,b, ~50-µm depth) and posterior stroma (Fig. 2c,d, ~350-µm depth). A visual inspection reveals the interwoven arrangement of the collagen fibers and the presence of two preferential orientations (PO).

A quantitative analysis of the stromal structure can be carried out by using the structure tensor (further information on this tool can be found in ref.^44^). This is a mathematical algorithm providing objective information on the degree of organization of the corneal stroma and the existing POs. As expected, the analysis of the SHG images of these healthy corneas presents a low structural dispersion (SD) which is associated to an organized distribution of collagen^44^. In particular, for the SHG images shown in Fig. 2a–d the SD values ranged between 14° and 22°.

For the sense of completeness Fig. 2e,f compare SHG images of the corneal stroma acquired in *in vivo* and *ex vivo* human eyes. It can be observed that both samples present a similar overall lamellar distribution, in particular a crosshatched pattern^21^, that is, an arrangement with collagen fibers running along two preferential directions. This simple observation is quantitatively shown in the PO histograms of Fig. 2g,h, computed using the structure tensor^44^. These two structural directions are associated to the two peaks of each plot.

The structure tensor used here has been reported to be useful to compare the spatial distribution of structural features in pairs of 2P images^45,46^. In addition, another comparison using the Mean Image Gradient (MIG) has also been calculated. MIG serves as edge detection operator to extract information from digital images^47^. The computed MGI values for the SHG images in Fig. 2e,f are 0.10 ± 0.06 and 0.09 ± 0.07 respectively. These values confirm that, although *ex vivo* 2P images seem much better resolved, the structural information provided by both types of images in terms of collagen structure is similar.

**Figure 3:**
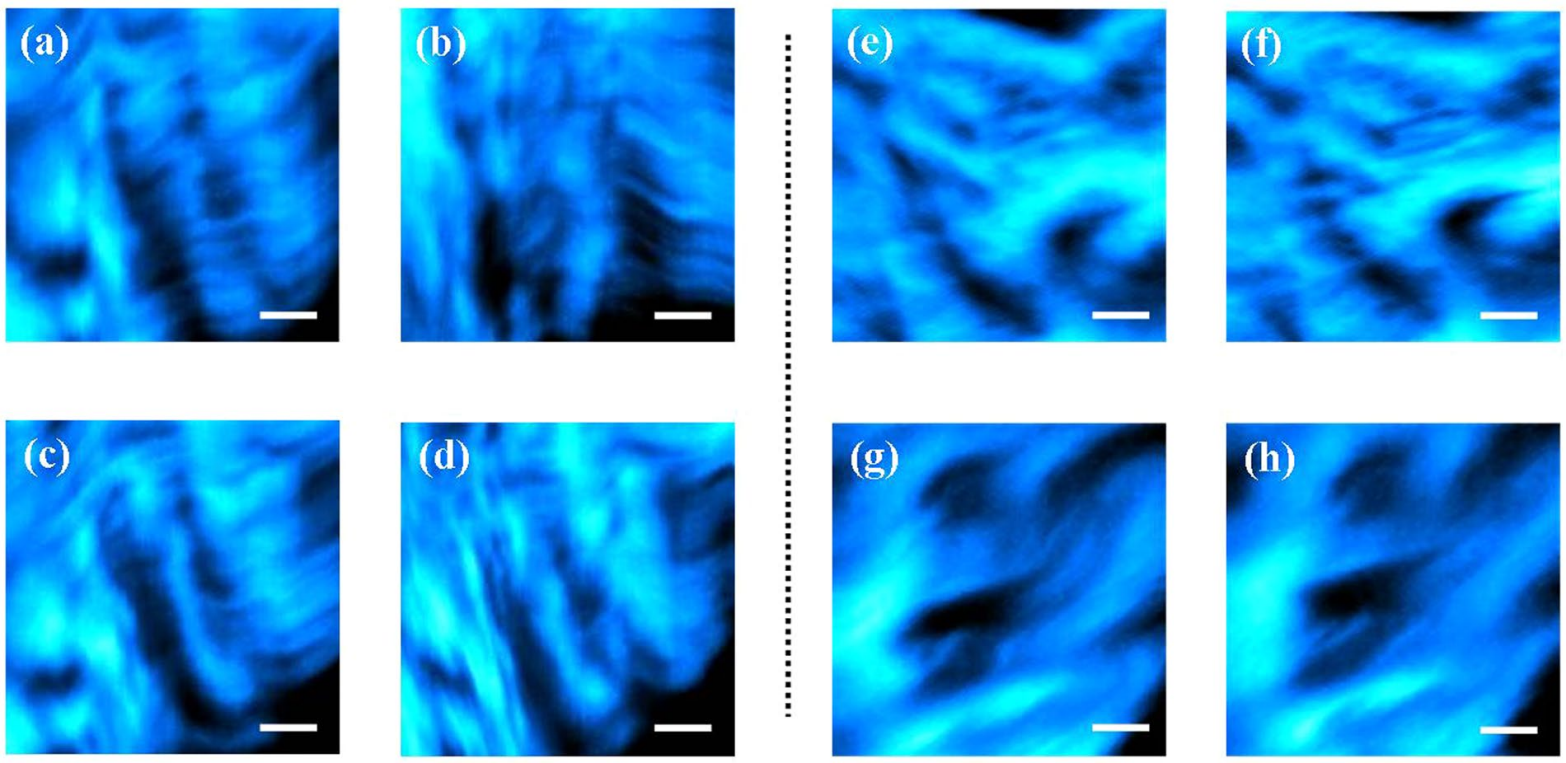
(**a**–**d**) SHG images of the living human sclera at two different depth locations spaced ~50-µm in subjects #1 (**a**,**c**) and #2 (**b**,**d**). (**e**–**h**) SHG images of the living human cornea (**e**,**f**) and sclera (**g**,**h**) recorded 2 s apart. Bar length: 50 µm.

SHG images of the sclera of both volunteers are depicted in Fig. 3a–d. Despite the opacity of the scleral tissue, two depth locations spaced 50 μm were imaged. The image resolution and the acquisition time were the same as in Fig. 2.

Despite a fixation target was used to minimize ocular movements (see Methods), the stability of the eye during measurements and the repeatability of the recorded images are crucial to test the accuracy and performance of the clinically-oriented instrument here developed. Figure 3e–h presents two pairs of *in vivo* SHG images (cornea and sclera) recorded 2 s apart. It can be observed how the recording operation is rapid enough to provide images with hardly noticeable movements. The differences in grey levels between pairs of SHG images were 2.4% and 1% for the top and bottom panels respectively. At this point it is interesting to note that all the images here shown are individual frames (i.e. “raw” images) without post-image processing procedures.

**Figure 4:**
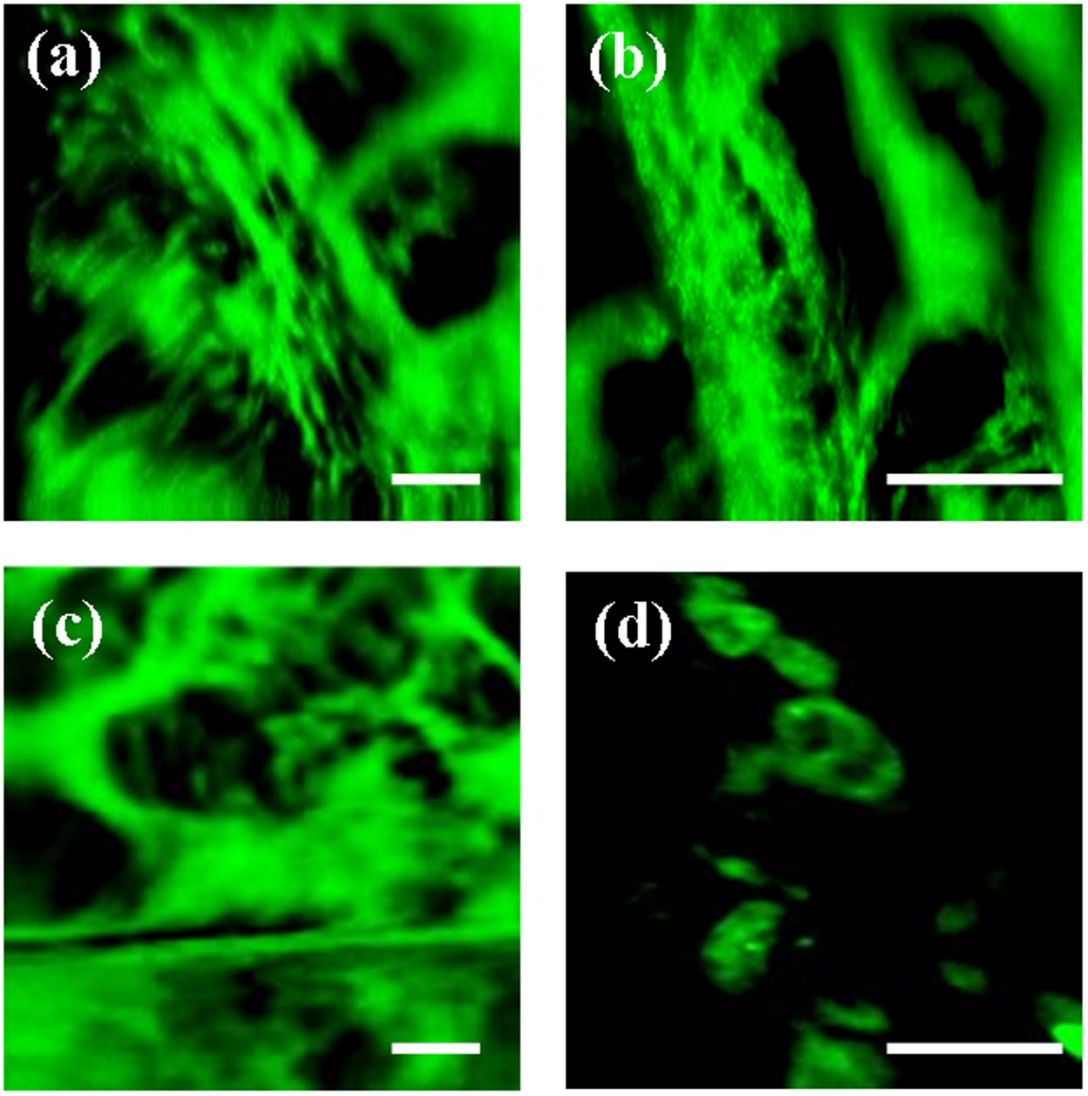
2P images (combining TPEF and SHG signals) of the trabecular meshwork region for subjects #1 (**a**,**b**) and #2 (**c**). (**d**) Shows details of the juxta-canalicular tissue. Scale bar: 50 µm.

2P images of the *in vivo* trabecular meshwork and the juxta-canalicular tissue are depicted in Fig. 4. As an illustrative example, the trabecular meshwork of subject #1 is shown for two different scanning areas (Fig. 4a,b). Figure 4c corresponds to subject #2. The juxta-canalicular tissue can be seen in Fig. 4d. To record these images, no filter was used in front of the detection unit. This means that the signal reaching the detector combines TPEF and SHG signals.

## Discussion

To the best of our knowledge, we have demonstrated 2P imaging in the intact human eye for the first time. A prototype was developed to successfully perform *in vivo* 2P images of different non-stained living ocular structures. This custom-made instrument is easy to operate and has a reduced size, an incident beam in a horizontal configuration and a fast scanning procedure. It might also be adapted to a clinical environment. Unlike clinical confocal instruments, our setup uses a long working-distance dry microscope objective. This avoids the contact with the eye and the use of ocular local anaesthetics, what greatly increases the subject’s comfort. Our results demonstrate that 2P images (individual frames, no image post-processing) can be obtained within the safety limits.

The performance of our instrument was tested by comparing living and *ex vivo* SHG images of the human cornea. The results showed a similar interwoven arrangement of the stromal lamellae. This is in agreement with previous findings^21^ and confirms an appropriate *in vivo* image acquisition.

Although with both experimental conditions lamellar domains were retrieved and similar structural information is obtained, living images seem to be “less sharp”, with features less outlined. The main reason is thought to be based on “motion artefacts”. Despite the exposure time and the image size are optimized to minimize these movements (apart from using the fixation test and the control cameras) there still exist remaining non-controlled ocular micro-movements that might contribute to this. It is worth to remember that 2P images here shown are “raw” images, that is, neither frame averaging nor image registration were employed here.

Zhang *et al*. reported 2P imaging of the cornea in the living mouse stained with injected fluorescent viable dyes^39^. Mice were anesthetized and a head-holding adapter was used to immobilize the animals. Moreover, a plastic eye cup filled with saline solution minimized heartbeat and breathing induced eyeball movement and fit the experimental requirements of the water-immersion microscope objective used. Since they only explored TPEF signals, the stroma fibers couldn’t be visualized and only cellular layers and some nerves were observed. Hao and co-authors couldn’t get 2P signal (neither TPEF nor SHG) from normal non-labelled *in vivo* rabbit corneas. The reason was thought to be the low laser power of the commercial instrument used, since they could detect TPEF signal within corneas injected with labelled cultured fibroblasts^37^. Lee *et al*. compared confocal and 2P microscopy in neovascularized mouse corneas. They visualized different corneal cells, but the collagen fibers in the stroma were not clearly showed^40^.

SHG imaging of anesthetized rat corneas has been reported by Latour and co-authors^38^. The use of a custom-built aplanation device minimized eye movements. In addition, an ophthalmic gel maintained the optical contact between the immersion objective and the eye. They did not provide precise data on the image acquisition time, but it was assumed that the main limitations referred to vital movements. To solve this issue, they performed image processing operations using small imaged regions (13 × 13 μm^2^) with a size larger that the cornea movement. Final images were of enough quality for the collagen lamellae to be visualized. However, despite those results, the authors stated that a few improvements in the acquisition conditions were required. In particular, they proposed a reduction of the acquisition time by increasing the pixel size (with the corresponding decrease in resolution), an improved immobilization of the animal’s eye and additional image processing to better compensate for eye motion. Although no alteration of the stromal collagen was observed during their experiments, they also claimed that further studies to calculate the maximum permissible power were necessary.

Most of the constraints of those previous studies involving animals were minimized or over passed in the present work. A precise evaluation of the femtosecond laser infrared light levels to avoid any tissue damage and ensure cell viability was presented herein. This ensured that our experimental conditions were well below the exposure limits. Although a test on the feasibility of safe *in vivo* TPEF imaging has been recently reported, that was exclusively centred on high resolution retinal imaging^48^.

Some of the issues associated with image stability have also been minimized here by establishing an appropriate imaging protocol. First, the subject’s head was immobilized by means of a customized chin-rest similar to that used in clinical instruments. The accurate positioning device of this chin rest, the control cameras and the collaboration of the subjects helped the operator to acquire images under optimal conditions. The subjects involved in the experiment felt comfortable during the entire image recording operation also due to the use of a non-immersion objective. Second, the fixation test made the subjects stare at a point to avoid large ocular movements. Finally, different experimental parameters involving safety limits, pixel dwell time, pixel-based image dimensions and imaged region size set an optimized acquisition time (see also Suppl. Material) to routinely record 2P images with minimal impact from ocular movements.

Despite some involuntary lateral eye movements might take place, 2P images were good enough to directly visualize collagen fibers. Axial ocular movements are expected to be marginal^38^ and do not disrupt the imaging procedure. Useful information on structural and morphological information can be extracted from the images. In particular, by combining SHG microscopy and the structure tensor analysis, is was possible to objectively identify the orientations of the collagen fibers. The size of the collagen fibers could also be computed and compared with previous *ex vivo* studies^21^.

Apart from collagen-based ocular structures, we were also able to image the trabecular meshwork. Images revealed small features (limbus ultra-structure and individual cells within the juxta-canalicular tissue) that might be important in the regulation of the intraocular pressure and glaucoma diagnosis. The *in vivo* appearance is in good agreement when compared to *ex vivo* images previously reported^15,16,49^.

An increase in contrast and/or resolution leads to better image quality. However, this implies image averaging (more sequential frames for the same imaged area) or an increase in the exposure time for each scanning position (i.e. higher photon integration time at detection unit). For *ex vivo* samples this is not an issue since the specimen is static. However, for *in vivo* measurements these are not appropriate approaches. Moreover, this would break the balance of the experimental acquisition conditions here reached.

2P images here shown are representative of normal healthy eyes. However, it has been shown that SHG microscopy is able to reveal structural changes produced by different corneal pathologies^23–32^, which seriously compromise its transparency and biomechanical properties. Effects of abnormal levels of intraocular pressure in the cornea, the sclera and the limbus could also be tracked with this technique. The characterization of the trabecular meshwork morphology as a function of the intraocular pressure will also help in glaucoma diagnosis. Since high myopia affects the scleral structure at different locations^50^, the visualization of spatial distribution of the collagen fibers would also be of great interest.

It is well-known that SHG signal predominantly occurs in the forward direction and the backward-emitted component is weaker^20,51^. As a result, SHG images acquired in both directions of propagation are qualitatively different^18,19^. Despite this, it has been reported that the retrieved lamellar orientation maps are the same for both modalities^11,38^. It is obvious that only the backward direction can be used with *in vivo* measurements.

In conclusion, real-time 2P image recording of the living human eye has been reported. A new clinically-oriented prototype was successfully developed for this goal. Images here showed enabled structural information of ocular structures without the use of markers. This minimally invasive method will open the door to *in vivo* investigations and would be crucial in ophthalmological environments. Diagnoses and follow-ups of different pathologies and surgeries will benefit from this approach in the next future.

## Methods

### Compact 2P microscope for living ocular measurements

**Figure 5:**
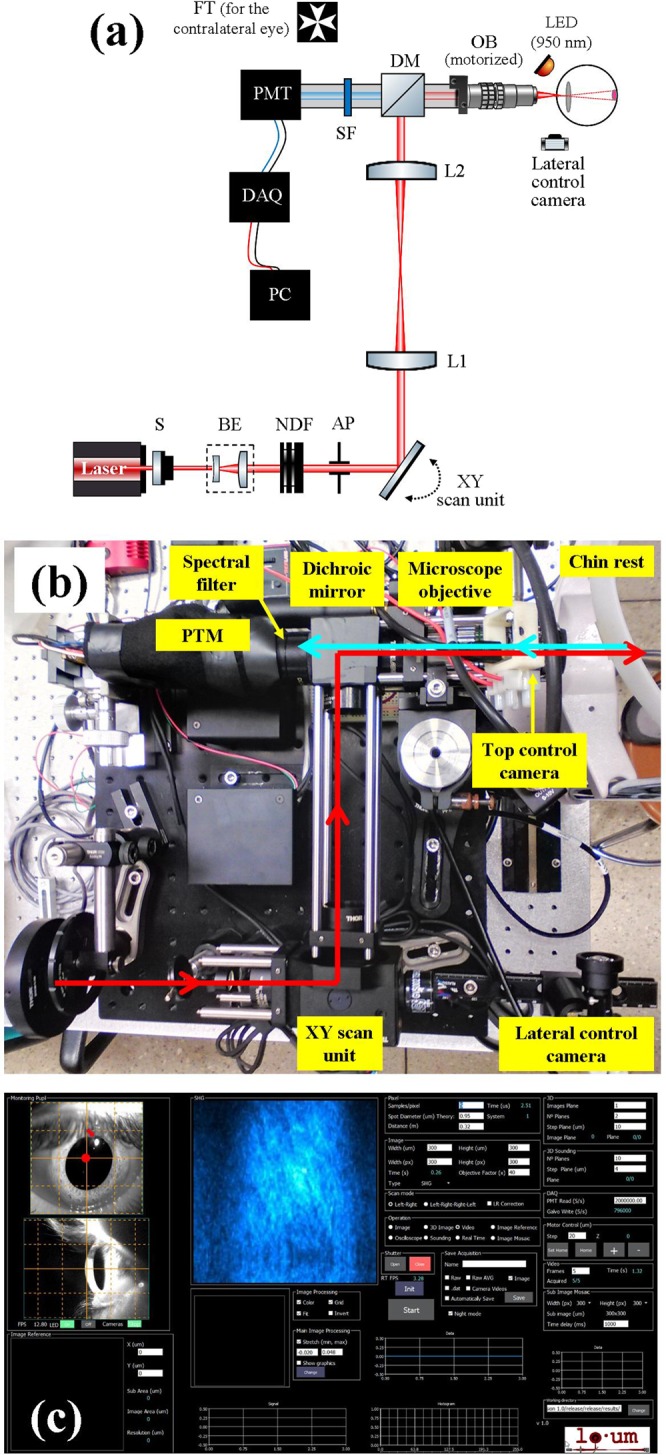
(**a**) Schematic diagram of the compact 2P microscope. S, shutter; BE, beam expander; NDF, neutral density filter; AP, aperture; L1 and L2, achromatic doublets; DM, dichroic mirror; OB, microscope objective; SF, spectral filter; PMT, photomultiplier tube; DAQ, data acquisition card; PC, computer. (**b**) Picture showing a top view of the actual instrument. The main elements are highlighted. (**c**) Customized user’s interface used for eye centration and image recording. The operator can choose the dimensions of the scanning area, the image size (in pixels) and the exposure time. For optical sectioning, the user must also set the number of planes and the axial distance between correlative planes.

A 2P microscope was built to fit a reduced space with dimensions 35 × 25 × 25 cm^3^. The design allowed living measurements in human eye. The prototype was composed of two platforms: the upper one contained all the optical elements (but the illumination laser system) and the bottom one included all the electronic components (data acquisition card, power supplies, connection board, etc). A picture and a schematic diagram of the device are shown in Fig. 5a,b.

The light source was a tunable Ti:Sapphire femtosecond laser (Mira 900 f, Coherent, St. Clara, CA, USA) set to a wavelength of 800 nm, with a repetition rate of 76 MHz. The pulse duration at the focal position of the microscope objective was 395 fs as measured with an auto-correlator (Mini, APE, Berlin, Germany). The light beam entered the microscope through an electro-mechanical shutter (S; SHB1, Thorlabs Inc., Newton, NJ, USA) and reached a Galilean telescope (BE) used to expand (2x magnification) and re-collimate the laser beam. The aperture AP fit the size of the beam to the back-aperture of the objective. The XY scanning was performed by means of a pair of mirrors attached to a dual-axis galvo unit (GVSM002, Thorlabs Inc., Newton, NJ, USA). After passing a telescopic system composed of two achromatic doublets (L1 and L2) and a dichroic short-pass mirror (DM, 69-218, Edmund Optics Ltd., York, UK), the beam entered the microscope objective OB (PLAN APO 20×, NA = 0.42, Edmund Optics Ltd., York, UK) and reached the living human eye under study. DM separated the excitation light from the generated 2P signal coming back from the eye. The objective was coupled to a motorized actuator (Z806, Thorlabs Inc., Newton, NJ, USA) to correct the focus position and provide optical sectioning along the Z direction through the human cornea (i.e. three-dimensional imaging). This microscope objective was chosen to have a long working-distance (WD = 20 mm). It provides non-immersion focusing, what avoids the uncomfortable eye-contact required in the actual clinical instruments such as corneal confocal microscopes^2^. The average laser power at the eye position was controlled by means of a neutral density filter (NDF) located behind the BE.

2P signal emissions (TPEF and/or SHG) from the eye were collected via the same microscope objective. They passed the corresponding spectral filter (SF) placed in front of the photomultiplier (PMT; H9305-02, Hamamatsu Photonics, Hamamatsu City, Japan) used as detector unit. The 2P signals were isolated by means of two different spectral filters: a band-pass for SHG (FB400-10, Thorlabs Inc., Newton, NJ, USA) and long-pass for TPEF (FELH0450, Thorlabs Inc., Newton, NJ, USA). An operational amplifier converted the output current from the PMT into voltage differences. A data acquisition card (DAQ; NI 6363, National Instruments, Austin, TX, USA) was used to synchronize the signal acquisition, the speed of the scanning unit, the shutter and the axial focus positioning. The microscope system was fully controlled via C ++ custom-written software.

Similar to a slit-lamp clinical device, this compact 2P microscope incorporates a 3-axis adjustable chin rest to ensure correct positioning and alignment of the eye, as well as comfort for the subject. In addition, to minimize ocular movements during imaging a Maltese cross was used as fixation test (FT). This test was retro-illuminated with a white diode and allowed the subject to stare at it with the non-measured contra-lateral eye.

The setup also included a dual camera system (DMM 42BUC03-ML, Imaging Source, Bremen, Germany) for the operator to control the correct position of the eye (as described below). One of the cameras (not shown in the figure) was placed on top of the objective and in front of the eye. The other control camera was set in a lateral position. To correctly visualize the position of the eye with respect to the incident beam, this was illuminated with infrared LEDs (950 nm). Once the position was reached, the LEDs were automatically turned off before the scanning imaging operation starts.

Figure 5c presents a screenshot of the user’s interface used to control the device and acquire 2P images of the living human eye. Left upper panel shows the image corresponding to the control front camera. The grid was calibrated to check the actual position of the laser spot on the ocular surface (the central cross with the red point on it served as a reference). The red arrow on the image indicates the 1st Purkinje, which is a result of the reflection of the LED placed in front of the corneal surface. The lateral camera controlled the axial position of the ocular surface, as shown in the bottom left panel. As an example, the panel on the right depicts a typical SHG image of the corneal stroma.

### Subjects and imaging protocol

**Figure 6:**
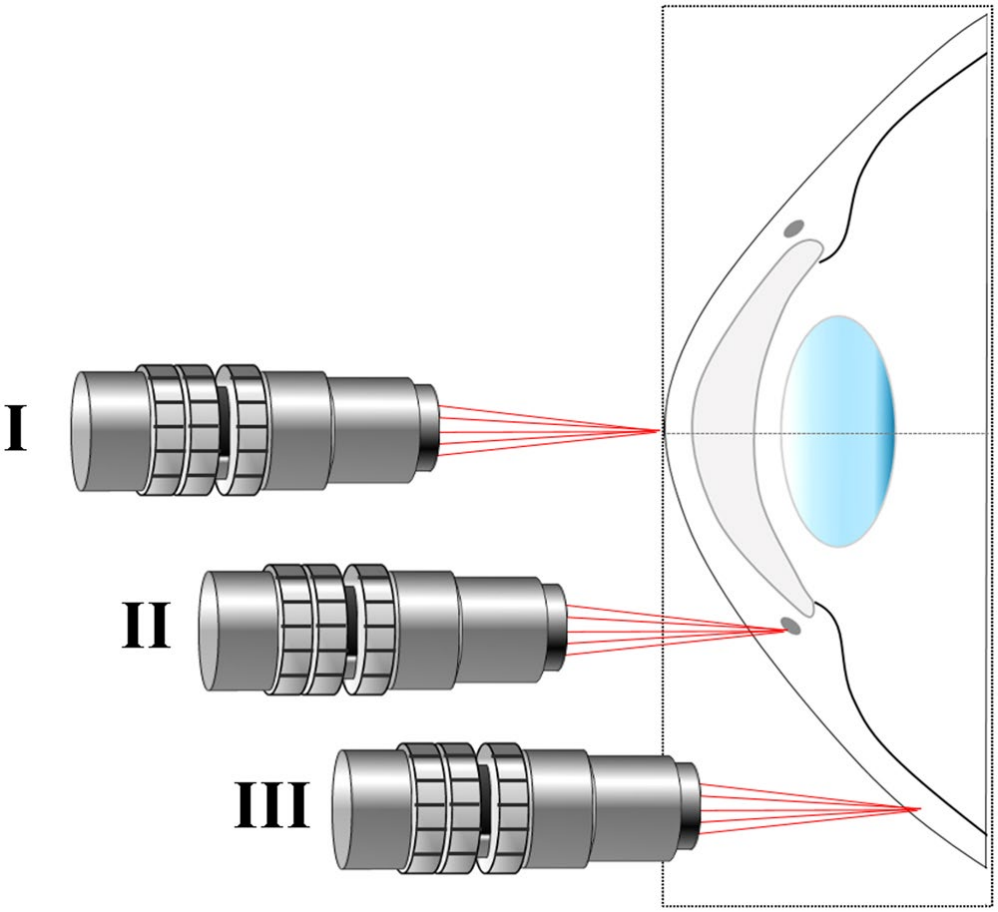
Simplified schematic indicating the locations of the imaged areas. It should be notice that this figure is intended for illustration, as the objective is unique and the eye rotates within its own orbit for the incident beam to be always perpendicular to the imaged region. For simplicity the eye’s rotation was omitted here.

Two healthy volunteers were involved in the present study. They were aged 34 (subject #1) and 47 (subject #2). 2P images corresponding to three different locations of the ocular structures were recorded (Fig. 6): corneal apex (labeled as location I), corneal limbus and trabecular meshwork tissue (location II) and sclera (location III). In this experiment, the maximum average laser power density at the corneal surface was 20 mW/cm^2^ (see more details on Suppl. Material). The image resolution was 1.5 µm/pixel (200 × 200 pixels) and the acquisition time 0.42 s. The entire protocol was approved by the Ethical Committee of the Universidad de Murcia, Spain. All experiments were performed in accordance with guidelines and regulations. Participants read the study participation informative document and signed the corresponding informed consent. *Ex vivo* human corneas from healthy donors (not suitable for transplantation) were provided by the Hospital Universitario Virgen de la Arrixaca, Murcia, Spain. This study and the experimental procedure were approved by the Ethical Review Board of the Hospital. Samples were treated following the instructions of the World Medical Association’s Declaration of Helsinki.

Once the appropriate laser power was set (by means of the NDF) and the imaging parameters established, the subject’s eye was centered and aligned using the chin rest, the control cameras and the Z-motor. Then, the user proceeded to initialize the image recording procedure through the interface. The synchronization of all the elements of the instrument makes the mechanical shutter close just after the image recording to avoid extra-laser exposure. If a 3D-scanning microscopy modality is chosen, the shutter is closed as the Z-motor moves between two consecutive axial locations (i.e. while the Z-motor moves from a plane to the next one).

## Supplementary information

SI Document

## Data Availability

The datasets and codes used within this paper are available from the corresponding author upon reasonable request.
