# Supplementary material for: In vivo two-photon microscopy of the human eye
Source: Sci Rep. 2019 Jul 12;9:10121. doi: 10.1038/s41598-019-46568-z (PMC6626016; doi:10.1038/s41598-019-46568-z)
Supplement: Supplementary file 1 — SI Document [file 41598_2019_46568_MOESM1_ESM.doc]

***In vivo* two-photon microscopy of the human eye**

**Francisco J. Ávila, Adrián Gambín, Pablo Artal and Juan M. Bueno***

Laboratorio de Óptica, Instituto Universitario de Investigación en Óptica y Nanofísica,

Universidad de Murcia, Campus de Espinardo (Ed.34), 30100 Murcia, Spain

* Corresponding author: bueno@um.es

**Supplementary Information**

***Calibration of the 2P imaging operation***

In a 2P microscopy experiment the quality of the images depends on the conversion efficiency of the nonlinear processes. Then, it is essential to calibrate the imaging procedure to set the experimental parameters in order to acquire 2P images. In the case of biological tissues this is very critical since these samples are more prone to photodamage and phototoxicity. In particular, the laser power density reaching the sample and the total acquisition time per image (or alternatively, the pixel dwell time) are the two essential parameters to have into account. This means that, during 2P image recording, a balance between the average density power and the exposure time is required. As a measure of the image quality, the Signal-to-Noise Ratio (SNR) has been used. This has been shown to be a useful image quality parameter to quantify images of ocular tissues^1S^.

Along the entire experiment (both calibration and *in vivo* measurements), the image size was set to 300x300 μm^2^ with 1.5 μm/pixel. With this resolution, corneal lamellae in ex-vivo human samples have been clearly visualized (see Ref. 21 of the main document).

For the imaging procedure calibration, an ex-vivo fresh porcine cornea was used as a sample (details on the sample’s manipulation can be found in Ref. 21 of the main document). This procedure consisted of two parts. As a first test, the dependence between the incident laser power density and the 2P signal was analyzed. For simplicity, an exposure time of 1 s was used. Although this time might be excessive for in vivo measurements, a general idea about the performance and accuracy of the imaging procedure can be obtained. The results are shown in Fig. S1a. As expected a quadratic dependence appears (R^2^=0.98, p=0.0028).

The second step was to determine the minimum laser power density and exposure time required to obtain a 2P image where physiological details can be seen. For this, series of images as a function of the exposure time and for different laser power values were acquired. The corresponding SNR was computed for each image. An example for a particular incident laser power density is depicted in Fig. S1b. It can be observed that at some temporal point, an increase in the image acquisition time hardly affects the SNR of the image. This calibration operation showed that an exposure time of 0.42 s combined with an average laser power density of 20 mW/cm^2^ were the optimum values to record 2P images where individual collagen fibers were visualized and the SNR value was around 2. Moreover, intensity profiles across the images recorded under these conditions showed that contrast of the fibers was at least 0.5 (data not shown).

This experimentally image rate here obtained agrees well with a previous 2P experiment in living (anesthetized) rats (see Ref. 38 of the main document). Although those authors did not provide much information, they claimed to have used a rate of a few tenths of a second per image. On the other hand, our imaging conditions provide a pixel dwell time of 10.5 μs. This value is also coherent with previous results in the literature that reported that a 2P image needs to be scanned within a time enough to ensure 10 pulses/pixel^2S^.”

**
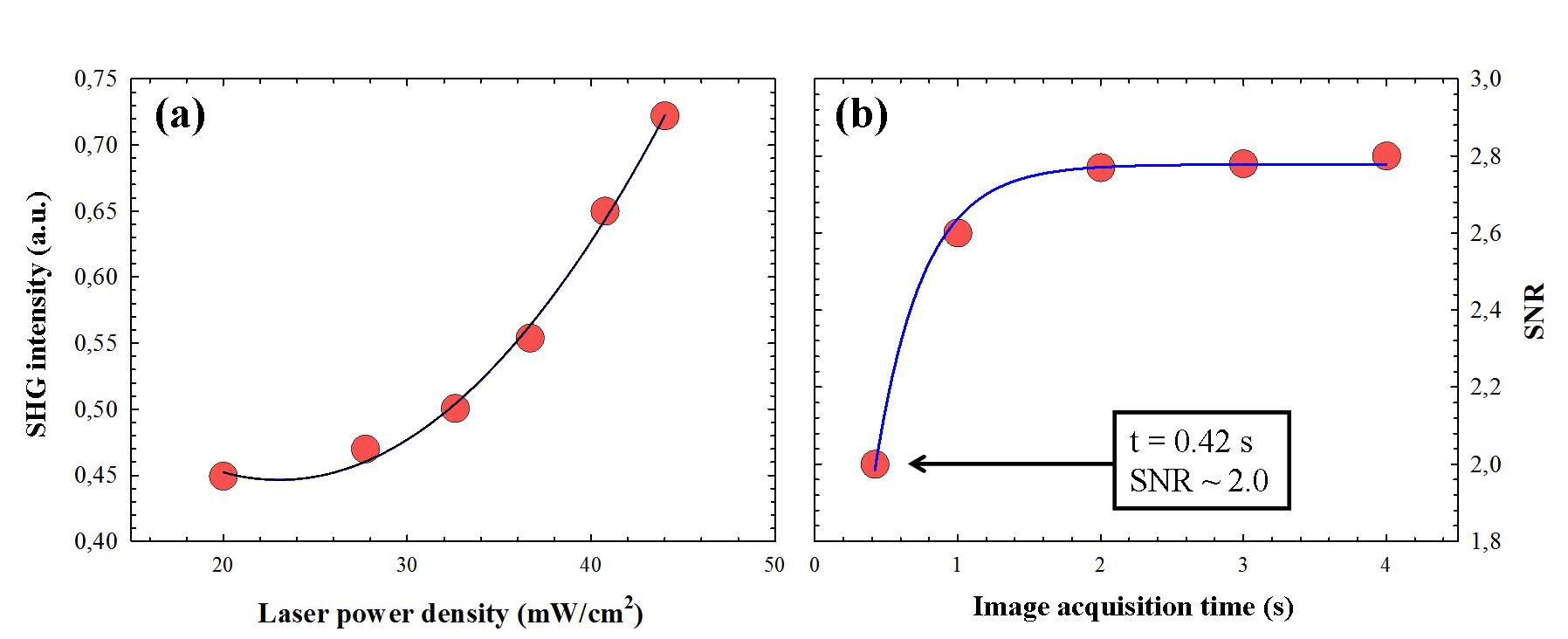
**

**Fig. S1**. Imaging procedure calibration. (a) SHG intensity as a function of the incident average laser power density for a fixed exposure time of 1 s. (b) SNR for different acquisition times for a particular incident laser power density (20 mW/cm^2^).

***References for this supplementary material***

[1S] Hunter, J.J., Cookson, C.J., Kisilak, M.L., Bueno, J.M. & Campbell, M.C. Characterizing image quality in a scanning laser ophthalmoscope with differing pinholes and induced scattered light. *J. Opt. Soc. Am. A*. **24**(5), 1284-1295 (2007).

[2S] Hoover, E.E. & Squier, J.A. Advances in multiphoton microscopy technology. *Nat. Photonics* **7**(2), 93-101 (2013).
